# An Exploratory Clinical Trial of a Novel Treatment for Giant Congenital Melanocytic Nevi Combining Inactivated Autologous Nevus Tissue by High Hydrostatic Pressure and a Cultured Epidermal Autograft: Study Protocol

**DOI:** 10.2196/resprot.6195

**Published:** 2016-08-11

**Authors:** Naoki Morimoto, Chizuru Jinno, Michiharu Sakamoto, Natsuko Kakudo, Tetsuji Yamaoka, Kenji Kusumoto

**Affiliations:** ^1^Department of Plastic and Reconstructive SurgeryKansai Medical UniversityHirakataJapan; ^2^Department of Plastic and Reconstructive SurgeryGraduate School of MedicineKyoto UniversityKyotoJapan; ^3^Department of Biomedical EngineeringNational Cerebral and Cardiovascular Center Research InstituteSuitaJapan

**Keywords:** giant congenital melanocytic nevi, cultured epidermal autograft, high hydrostatic pressurization, inactivation

## Abstract

**Background:**

Giant congenital melanocytic nevi (GCMNs) are large brown to black skin lesions that appear at birth and are associated with a risk of malignant transformation. It is often difficult to reconstruct large full-thickness skin defects after the removal of GCMNs.

**Objective:**

To overcome this difficulty we developed a novel treatment to inactivate nevus tissue and reconstruct the skin defect using the nevus tissue itself. For this research, we designed an exploratory clinical study to investigate the safety and efficacy of a novel treatment combining the engraftment of autologous nevus tissue inactivated by high hydrostatic pressurization with a cultured epidermal autograft (CEA).

**Methods:**

Patients with congenital melanocytic nevi that were not expected to be closed by primary closure will be recruited for the present study. The target number of nevi is 10. The full-thickness nevus of the target is removed and pressurized at 200 MPa for 10 minutes. The pressurized and inactivated nevus is sutured to the original site. A small section of the patient’s normal skin is taken from around the nevus region and a CEA is prepared after a 3-week culturing process. The CEA is then grafted onto the engrafted inactivated nevus at four weeks after its retransplantation. The primary endpoint is the engraftment of the CEA at 8 weeks after its transplantation and is defined as being engrafted when the engraftment area of the inactivated nevus is 60% or more of the pretransplantation nevus area and when 80% or more of the transplanted inactivated nevus is epithelialized.

**Results:**

The study protocol was approved by the Institutional Review Board of Kansai Medical University (No. 1520-2, January 5, 2016: version 1.3). The study opened for recruitment in February 2016.

**Conclusions:**

This protocol is designed to show feasibility in delivering a novel treatment combining the engraftment of inactivated autologous nevus tissue and CEA. This is the first-in-man clinical trial of this treatment, and it should be a promising treatment of patients suffering from GCMN.

**Trial Registration:**

University Hospital Medical Information Network: UMIN000020732; https://upload.umin.ac.jp/cgi-open-bin/ctr_e/ctr_view.cgi?recptno=R000022198 (Archived by WebCite at http://www.webcitation.org/6jLZH2vDN)

## Introduction

Giant congenital melanocytic nevi (GCMNs) are large brown to black skin lesions that appear at birth and have a diameter of more than 20 cm [1-5]. GCMNs, which are reported to occur in approximately 1 in 20,000 newborns [1,2], are associated with a risk of transformation that usually results in malignant melanoma. The incidence of malignant transformation into malignant melanoma is reported to be 0.7% to 8.2% [2,5,6]. Histologically, nevus cells are present throughout the entire layer of the dermis and, in some cases, the subcutaneous tissue. Thus, the removal of the full thickness of the nevus tissue is necessary to remove nevus cells completely and to prevent the emergence of melanoma [1-5]. It is often difficult to reconstruct large full-thickness skin defects after the removal of GCMNs. It is also reported that melanoma and aneurocutaneous melanocytosis are most likely in patients with GCMNs that have a final size of more than 40 cm in diameter with numerous satellite nevi [6]. Thus, the skin reconstruction of GCMNs remains a challenge in the field of plastic and reconstructive surgery.

We have already reported that all cells existing in the human skin, porcine skin, and nevus tissue were inactivated completely after high hydrostatic pressurization (HHP) at pressures of more than 200 MPa [7,8]. We also reported that the human-cultured epidermis took and survived on the pressurized skin and nevus after pressurization at 200 MPa [7,9]. Skin consists of the epidermis, which acts as a barrier against infection and water loss, and the dermis, which supports and supplies nutrition to the epidermis. Regarding skin reconstruction, the regeneration of the epidermis by a cultured epidermal autograft (CEA) using Green’s method was established in the 1970s [10]. A method of dermal regeneration that achieves sufficient strength and elasticity has not yet been established [11,12].

We designed an exploratory clinical study to investigate the safety and efficacy of a novel treatment combining autologous nevus tissue inactivated by HHP and a CEA. This trial is the first-in-man clinical trial to reuse and apply autologous nevus inactivated by HHP to reconstruct autologous dermis after the removal of the nevus itself without discarding the nevus tissue. JACE (Japan Tissue Engineering Co Ltd, Gamagori, Japan) is a CEA product prepared using Green’s method that has been approved for use in Japan since 2007 [13]; CEA products are approved for use in other countries as well. A CEA of sufficient size to cover the total surface area can be produced from an autologous skin specimen of approximately 1 cm × 2 cm in size. This treatment can therefore be expected to overcome the issues of GCMN treatment and prevent the malignant transformation of GCMNs.

## Methods

### Primary Objective

The objective of this study is to evaluate the safety and efficacy of a novel treatment combining autologous nevus tissue inactivated by HHP with CEA.

### Design

This study is an open-label, nonrandomized, single-arm, controlled clinical trial that is a prospective study using historical data as a control. All of the patients will receive a combination treatment consisting of autologous nevus tissue inactivated by HHP with CEA.

The take rate of the CEAs implanted on the inactivated nevus tissue will be estimated at 8 weeks after CEA implantation and compared to the historical data of the take rate of CEAs. This comparison will provide useful information for designing and conducting future trials.

### Setting and Participants

Patients with congenital melanocytic nevi will be identified by physicians in Osaka and other prefectures who will refer them to this study, being conducted at Kansai Medical University Hirakata Hospital.

Patients 7 months of age and older who are able to undergo surgery under general or local anesthesia and can give informed consent (proxy consent permitted for pediatric patients only) are included. Patients must have a congenital melanocytic nevi that is not expected to be closed by primary closure (patients require a skin graft or skin flap surgery to close the skin defect after its removal) with a target pigmented nevus area of 0.25% or more of the total body surface area (1/4 of the palmar area, including the fingers, taking the palm including the fingers as 1% of the total body surface area).

Exclusion criteria are extensive scarring from previous therapies (in whom the engraftment of the inactivated nevus is not expected), previous treatment of the target site by CEA in other clinical studies, history of malignant tumors with a disease-free interval of 5 years or less, two previous protocol treatments from this study, or patients who are judged by the investigator or subinvestigator to be inappropriate as study subjects.

### Interventions

#### Removal of the Target Nevus and Its Inactivation by HHP

The full thickness nevus of the target is removed under general or local anesthesia and the subcutaneous adipose tissue is removed by scissors and packed into a polyethylene bag filled with normal saline solution (Otsuka Pharmaceutical Co Ltd, Tokyo, Japan) and sealed. We use a portable HHP device that was developed in collaboration with Kitaoka Iron Works Co Ltd (Osaka, Japan) [14]. The polyethylene bag containing the resected nevus is then placed in the cavity of the cell of this HHP device and the cavity is filled with distilled water (Otsuka Pharmaceutical Co Ltd, Tokyo, Japan). The pressure is increased up to 200 MPa and maintained for 10 minutes, then decreased to atmospheric pressure over a period of a few seconds.

#### Retransplantation of the Inactivated Nevus

The pressurized and inactivated nevus is taken from the polyethylene bag and sutured to the original site. The nevus is fixed and stabilized to the wound bed using a tie-over dressing or negative pressure wound dressing for about one week after the retransplantation, as would be performed in a usual skin graft procedure. The graft is then covered by wound dressings or ointment gauze until the application of CEA. The inactivated nevus does not have any cellular components; however, within a few weeks fibroblasts and capillaries will infiltrate the nevus and the engrafted nevus will serve as an autologous dermis without nevus cells and will serve as a recipient floor for the CEA.

#### Preparation and Application of the CEA

A small section of the patient’s normal skin (approximately 1 cm × 2 cm) is taken from around the nevus region for the preparation of the CEA. This skin is transported to a laboratory and the CEA is prepared after a culturing process that usually takes three weeks. The CEA will be grafted on the engrafted inactivated nevus at four weeks after the retransplantation of the inactivated nevus.

#### Subsequent Therapy

The inactivated nevus and CEA is treated in the same way as a skin graft. The use of ointments and wound dressings are allowed locally and no particular restrictions will be imposed during the study period.

#### Digital Photography for the Assessment of Healing

A digital camera is used to capture images of the grafts with a calibrator (ColorChecker Passport, X-lite Inc, Michigan, United States) placed on the skin adjacent to the wound. The color and size of the images can be adjusted using the ColorChecker Passport and an image editing software program (Adobe Photoshop, Adobe Systems Inc) to assess the area and color of the target nevus. As with the primary endpoint, the nevus evaluation committee members will assess the original area of the nevus before its removal and the engrafted area of the pressurized nevus and epithelized area on the pressurized nevus at 8 weeks after transplantation of the CEA.

### Primary Endpoint

The primary endpoint is engraftment of the CEA at 8 weeks after its transplantation. The CEA is considered to be engrafted when the engraftment area of the inactivated nevus is 60% or more of the pretransplantation nevus area and 80% or more of the transplanted inactivated nevus is epithelialized. The proportion of the engraftment area is referred to as the engraftment rate. The engraftment assessment is carried out by three reviewers of the nevus evaluation committee. Macroscopic pictures of the grafts will be provided to each reviewer without patient information. The reviewers will determine the engraftment area of the inactivated nevus and the epithelized area based on these photographs.

With regard to the cutoff point of 60%, it has been reported that when autologous skin grafting is performed, the area of the skin graft contracts to approximately 80% at 3 months after grafting. Furthermore, in some cases the graft is reported to contract to approximately 60% [15]. We took the above information into consideration and set the cutoff point at 60% or more. Cases in which the engrafted area falls below 60% are not considered to be engrafted even if they are mostly epithelialized. Damage to the inactivated nevus by HHP may lead to such a degree of contracture.

When 80% or more of the CEA is epithelialized, it may be considered to be almost completely epithelized at the macroscopic level. Erosion and blister formation or shedding may occur in the early phase following grafting. The cutoff value for epithelialization was therefore set at 80% or more. This value takes the possibility of erosion and shedding, which may occur in some cases, into consideration [16].

### Secondary Endpoints

The event name, grade, and outcome of adverse events and adverse reactions will be assessed according to the Common Terminology Criteria for Adverse Events version 4.0. Adverse events and adverse reactions occurring from the day of the transplantation of the inactivated nevus until the end of follow-up will be assessed.

A secondary endpoint is engraftment of the inactivated nevus at 4 weeks after its transplantation. The inactivated nevus is considered to be engrafted when the pressurized nevus forms a successful recipient floor for the CEA.

### Blinding

The baseline nevus area before its removal, the engrafted area of the inactivated nevus, and the epithelized area 8 weeks after CEA transplantation will be independently estimated by blinding the members of the nevus evaluation committee. The patients will be unblinded, and the transplantation of the inactivated nevus and CEA will be performed by unblinded investigators.

### Sample Size

The target number of grafted sites of the inactivated nevus is 10 sites. In this study, one patient can receive the study treatment for up to 2 sites, thus a minimum of 5 patients will be included in this study.

The subjects of this study include pigmented nevus patients for whom primary closure is not expected. Regarding the number of newly diagnosed GCMN cases each year, few such cases are diagnosed at Kansai Medical University Hirakata Hospital, while approximately 5 cases are diagnosed at Kyoto University Hospital (which treats more cases). Considering the number of patients with a history of treatment for residual nevi and the need to include 10 sites (at least 5 cases), we determined that the enrollment period should be 1 year and 6 months.

### Study Schedule

**Figure 1:**
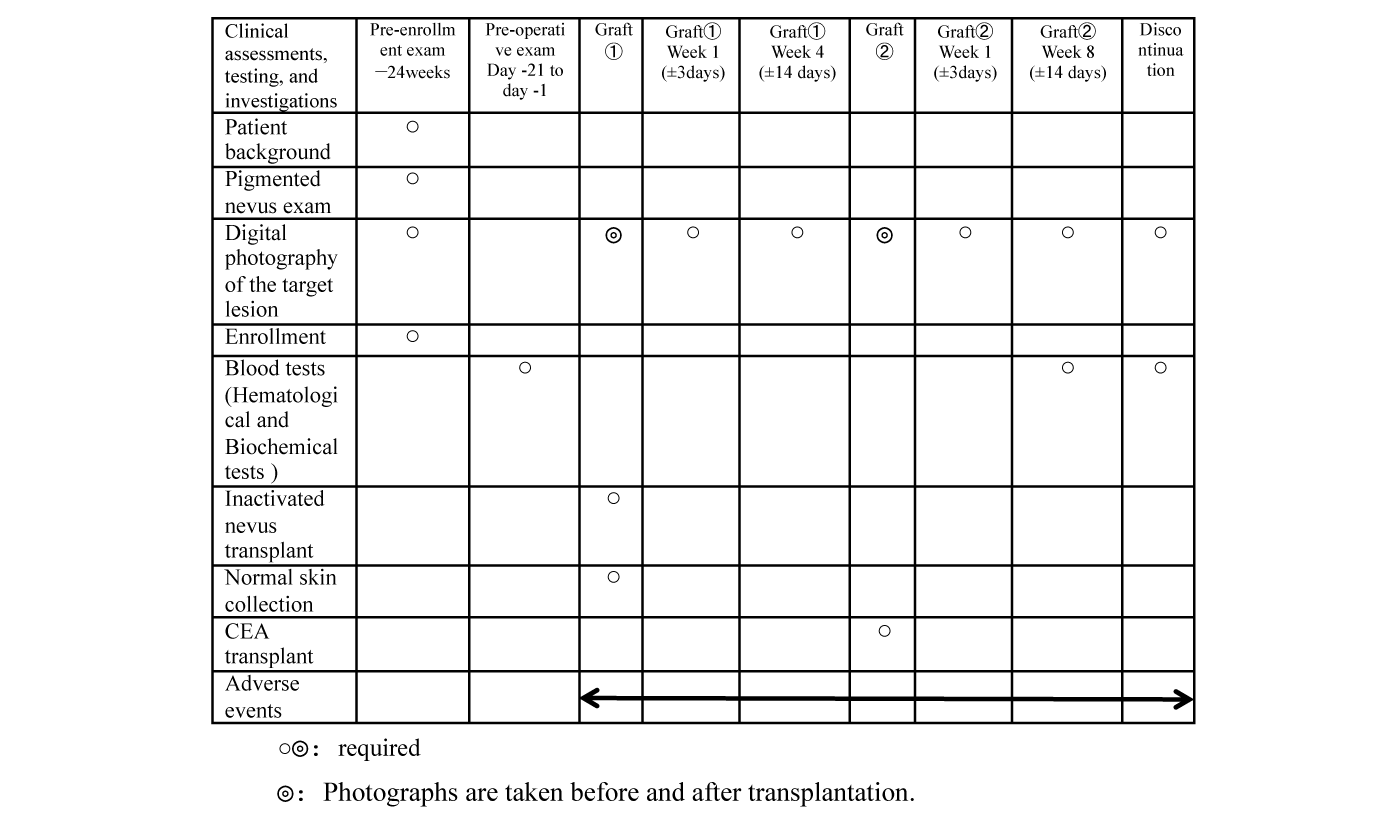
Schedule of the study assessments and evaluations.

The schedule of the study assessments and evaluations is shown in Figure 1. The study period will be from the day of informed consent to 12 weeks after the transplantation of the inactivated nevus. The data for evaluating the efficacy and safety of this study will be collected at enrollment, day 1, weeks 1 and 4 after transplantation of the inactivated nevus, and weeks 1 and 8 after the transplantation of the CEA.

### Statistical Analysis

When CEAs are transplanted on a wound bed without autologous dermal tissue, the engraftment rate is reported to be only 20%. In contrast, the engraftment rate is expected to be 80% when the CEA is transplanted on dermal tissue [17]. In this study, an engraftment rate of 80% can be expected because the autologous dermis is reconstructed using inactivated nevus tissue.

It was considered that the treatment could be deemed effective if it exceeds this engraftment rate. In this case, with an α error (one-sided) of .05 and a statistical power of 90%, the number of sites required was calculated to be 6 sites. Taking into consideration the possibility that some cases may be excluded from the analysis, we considered that there should be 10 target sites.

In our study, a single patient may undergo treatment for up to 2 sites. When two treatments are performed for the same patient, the first and second surgical sites are regarded as independent sites whether they are separated from each other by normal skin or nevus skin. Moreover, when providing simultaneous treatment to multiple sites in one subject, the largest treatment site will be included in the statistical analysis.

The incidences of adverse events and adverse events that can be causally related to the inactivated nevus and CEA application will be evaluated based on the event and severity. An interim analyses and auditing will not be planned; however, the investigators will monitor adverse events and other unintended effects of the trial interventions. A data monitoring committee consisting from three independent clinicians will also monitor patient safety and have the power to recommend termination of the study based on the evaluation of these results.

### Ethics

This study is being conducted in compliance with the International Council for Harmonisation Good Clinical Practice and in accordance with the latest revision of the Declaration of Helsinki, Pharmaceutical Affairs Law and all applicable Japanese laws and regulations, as well all local laws and regulations and all applicable guidelines. This protocol and any amendments received Institutional Review Board (IRB) approval from Kansai Medical University (Approval number 1520-2).

### Subject Consent

Informed consent will be obtained from all potential study participants using an IRB-approved informed consent form. The clinical investigator will inform the potential study subject about all of the pertinent aspects of the study. The subject must sufficiently understand the content of the information form before providing written consent. The consent form must be dated and signed by both the investigator and the participant. The subjects are also informed that their medical care will not be affected if they do not choose to participate in this study. The consent forms will be retained at Kansai Medical University Hirakata Hospital and the information form and a copy of the consent form are handed to the participant. Whenever the investigator obtains information that may affect the participant’s willingness to continue participation in the study, the investigator or subinvestigator will immediately inform the participant and record this observation and subsequently reconfirm the participant’s willingness to continue to participate in the study.

### Dissemination

The findings of this trial will be disseminated through peer-reviewed journals and national and international scientific meetings as well as to the patients.

## Results

The study protocol was approved by the IRB of Kansai Medical University (No. 1520-2, January 5, 2016: version 1.3). The study opened for recruitment in February 2016, and recruitment will continue until August 2017.

## Discussion

This study was designed to address the safety and efficacy of the engraftment of nevus tissue inactivated by HHP for dermal reconstruction in combination with CEA. This study is the first-in-man clinical trial to reuse the nevus tissue inactivated by HHP as an autologous dermis for the reconstruction of full-thickness defects after the inactivation of the pigmented nevus. As for the regeneration of the dermal component for the recipient site for the CEA, an allogeneic skin graft is the first line of the treatment. However, the supply of allogeneic skin is limited and constantly insufficient in Japan. Allogeneic skin accelerates the formation of the dermal component, but it does not survive on the recipient site nor does it serve as an autologous dermis [13]. The inactivated nevus is the patient's autologous tissue, and it has the potential to survive and serve as an autologous dermis without inflammation or rejection.

HHP technology can inactivate all of the cells in the nevus tissue, regardless of the thickness of the nevus tissue. According to Pascal's principle, pressure is transmitted, undiminished, in an enclosed static fluid. However, the melanin pigment is not damaged after HHP and it will remain after retransplantation. We think that the melanin pigment will be gradually biodegrade because it is a peptide and the nevus cells that produce the pigment will not be present after HHP. If the pigment does not biodegrade, we will use laser treatment to remove the pigment that remains after the present protocol.

This HHP treatment seeks to inactivate tumor cells in the tumor tissue and reuse the matrix itself after inactivation. This concept could be applied to the treatment of other skin tumors that require extensive safety margins such as malignant melanoma or squamous cell carcinoma. In addition, this could also be used in the treatment of tumors of other types of tissue. If successful, this HHP technology could lead to breakthrough treatments for benign and malignant tumors from the perspective of tissue regeneration.

## Abbreviations

CEA: cultured epidermal autograft
GCMN: giant congenital melanocytic nevus
HHP: high hydrostatic pressurization
IRB: institutional review board
